# Terminus-specific antibody development and bioanalytical assay validation to quantify active DR10624 in pharmacokinetic studies

**DOI:** 10.3389/fphar.2026.1863402

**Published:** 2026-08-03

**Authors:** Zhenxing Zhou, Kexin Cao, Gaofeng Yao, Yuanyuan Liu, Wenwen Duan, Chengcheng Yang, Shimei Sheng, Yanshan Huang

**Affiliations:** Department of Innovative Drug Discovery and Development, Zhejiang Doer Biologics Co., Ltd., Hangzhou, China

**Keywords:** DR10624, immunoassay, method validation, multi-specific drug, pharmacokinetics

## Abstract

**Introduction:**

Multi-specific drugs play important roles in addressing multi-factorial disease conditions. DR10624 is a first-in-class long acting Fc fusion triple agonist targeting FGF21R, GLP-1R, and GCGR, which has shown promising efficacy in reducing liver fat and triglycerides. Accurate quantification of the intact, pharmacologically active form of DR10624 in serum is essential for reliable pharmacokinetic (PK) characterization.

**Methods:**

Rabbit monoclonal antibodies that specifically recognize the N- and C-termini regions of DR10624 were developed and strategically paired for precise quantification. An electrochemiluminescence (ECL) based immunoassay was established and rigorously validated in accordance with regulatory guidelines. Additionally, a preclinical pharmacokinetic study in cynomolgus monkeys was performed to compare drug exposure using different assay formats.

**Results:**

Antibody screening successfully identified highly specific rabbit monoclonal antibodies, notably clones 4G7 and 1A12, which selectively bound to the intact N- and C-termini of DR10624 and effectively neutralized its GLP-1/GCG and FGF21 activities, respectively. Furthermore, the preclinical pharmacokinetic study in cynomolgus monkeys revealed a marked discrepancy between total and intact DR10624 exposure, indicating *in vivo* instability and underscoring the need to quantify active drug concentrations. The ECL-based immunoassay was successfully validated across a wide concentration range of 5.00–1,250 ng/mL in serum, confirming its accuracy, precision, selectivity, linearity, absence of hook effect, and stability.

**Discussion:**

The validated dual-monoclonal ECL method provides a robust and specific approach for quantifying intact, bioactive DR10624 in human serum. This assay supports reliable PK assessment in ongoing and future clinical trials, facilitating the continued development of DR10624 for the treatment of MASLD/MASH, severe hypertriglyceridemia (SHTG), and related metabolic diseases.

## 1 Introduction

The traditional paradigm of “one molecule, one target, one disease” has achieved success in treating certain conditions; however, it often proves inadequate for addressing multi-factorial diseases such as cancer, neurodegenerative disorders, cardiovascular diseases, diabetes, and infections (Talevi, 2015). These complex diseases involve multiple receptors or signaling pathways, and interventions focusing on a single target frequently fail to restore physiological balance due to compensatory mechanisms and functional redundancies within biological systems. Multi-specific drugs, also referred to as multi-functional drugs, or multi-target drugs, are single chemical entities designed to modulate several molecular targets simultaneously (Zhou et al., 2019; Li et al., 2021; Deshaies, 2025; Deshaies, 2020). Over the past decade, this approach has attracted considerable interest as a promising therapeutic strategy for complex diseases. Nevertheless, the development of multi-specific drugs poses numerous bioanalytical and methodological challenges throughout all stages of drug discovery and development (Bolognesi, 2013). These challenges stem from the inherently complex structures, which contain multiple functional domains, and from complications in the accurate characterization of their physicochemical properties. Such structural complexity leads to intricate *in vivo* metabolic behavior, making it more challenging to evaluate the relationship between pharmacokinetics (PK) and pharmacodynamics (PD).

Fibroblast growth factor 21 (FGF21), glucagon-like peptide-1 (GLP-1), glucose-dependent insulinotropic peptide (GIP), and glucagon (GCG) are hormones with significant endocrine effects, playing crucial roles in metabolic homeostasis, particularly in glucose and lipid metabolism (Harrison et al., 2024; Brouwers et al., 2024). FGF21 exerts its action through a receptor complex comprising a co-receptor, β-klotho (KLB), and a canonical FGF receptor (FGFR), predominantly FGFR1c, FGFR2c, and FGFR3c (Yie et al., 2012; Suzuki et al., 2008). FGF21 engages with KLB and FGFR isoforms via its C-terminal and N-terminal sequences, respectively, initiating intracellular signaling cascades. Previous studies have shown that truncation of either the C-terminal or N-terminal of FGF21 impairs, or even abolishes, its agonistic efficacy and potency, underscoring the importance of structural integrity for its function (Yie et al., 2009; Micanovic et al., 2009). GLP-1, GIP, and GCG mediate their effects by binding to specific G protein-coupled receptors (GPCRs): GLP-1R, GIPR, and GCGR (Lamb et al., 2025; Li et al., 2024; Araki et al., 2022), respectively. Dipeptidyl peptidase IV (DPP-IV) plays a critical role in modulating the incretin system, as it cleaves the N-terminal dipeptide from GLP-1, GCG, and GIP, rendering them inactive. Modifications to the N-terminus of these peptides can enhance their resistance to DPP-IV, thereby prolonging their action (Mishriky et al., 2019; Deacon, 2019; Gault et al., 2013).

The combination of Fibroblast Growth Factor 21 (FGF21) and incretins, particularly Glucagon-like Peptide-1 (GLP-1), is a promising therapeutic strategy aimed at harnessing the complementary actions of these two metabolic hormones to treat complex metabolic diseases like type 2 diabetes (T2D), obesity, and metabolic dysfunction-associated steatohepatitis (MASH) (Pan et al., 2021; Chui et al., 2024). In a Phase 2b trial (SYMMETRY Cohort D), patients with MASH and T2D treated with Efruxifermin **(**an Fc-FGF21 analog, also known as AKR-001) as an add-on to existing GLP-1RA (GLP-1R agonist) treatment showed a 65% reduction in liver fat, dramatically exceeding the 10% decrease seen in the GLP-1RA-alone group (Carvalho, 2023). This combination also suggested the potential to accelerate the regression of fibrosis, as indicated by a significant reduction in the non-invasive marker ELF (the non-invasive marker of fibrosis) score. C-192, a tetra-specific agonist designed to simultaneously target four receptors, including GLP-1R, GCGR, FGF21 receptor complex and IL-1R1 (Interleukin-1 receptor 1), was highly effective in treating hepatic steatosis and inflammation, leading to complete recovery in all tested mice (Kim et al., 2023). All these findings indicate that FGF21-based combination therapy, particularly with incretins receptor agonists, represents a promising future direction for comprehensive treatment of T2D, obesity, and MASH.

DR10624 is a first-in-class (FIC) long-acting Fc-fusion triple agonist. It consists of a mutated FGF21 moiety at the C-terminus and a hybrid peptide with dual agonistic activities targeting GLP-1R and GCGR at the N-terminus. In non-clinical studies, DR10624 has demonstrated notable efficacy in reducing body weight, normalizing blood lipids levels, and improving glycemic control, positioning its potential as a therapeutic candidate for MASH (Fang et al., 2025a). In a Phase 2a clinical trial involving subjects with modest hypertriglyceridemia (HTG), once-weekly administration of DR10624 over 12 weeks led to significant improvements in key metabolic parameters (Fang et al., 2025b). In the 50 mg dose group, 100% of subjects achieved a ≥50% relative reduction in liver fat, along with a 70.2% reduction in triglycerides (TG). These outstanding improvements in lipid profile and liver fat further highlight DR10624 as an innovative and promising treatment for metabolic disorders.

Non-human primate (NHP) species, including cynomolgus macaque and rhesus macaque, are frequently recognized as the most relevant species and are common animal models for pharmacokinetic (PK) studies characterizing human therapeutics, due to their genetic and physiological similarities to humans (Chapm et al., 2010). In essence, the PK study in NHP serves as a highly relevant model in preclinical drug development—functioning as a critical bridge between laboratory experiments and clinical trials by characterizing how a drug is processed in a primate system and helping in estimating the maximum recommended starting dose (MRSD) for first-in-human (FIH) clinical trials (Tseng et al., 2015).

*In vitro*, cell-based potency assays showed that DR10624 variants lacking the first two N-terminal residues (ΔN2) or the last four C-terminal residues (ΔC4) each completely lost agonist activity. Because bioactivity depends on the structural integrity of both termini, precise quantification of the intact molecule is essential for pharmacokinetic and exposure–response assessments in clinical studies. We therefore generated rabbit monoclonal antibodies targeting the native N- and C-terminal regions of DR10624 using a rational immunogen design and screening strategy. Antibody 4G7, which recognizes the intact N terminus, and antibody 1A12, which recognizes the intact C terminus, were paired to ensure selective measurement of pharmacologically active, intact DR10624. In cynomolgus monkeys, a preclinical pharmacokinetic study revealed a marked discrepancy between total and intact DR10624 exposure, indicating *in vivo* instability and underscoring the need to quantify active drug concentrations in subsequent long-term, repeat-dose toxicity studies in NHPs and in clinical investigations. Building on these findings, we developed a highly sensitive electrochemiluminescence (ECL) immunoassay using the 4G7/1A12 pair to quantify intact DR10624 in human serum, validated the method in accordance with regulatory bioanalytical guidance, and applied it in the clinical study of DR10624 (NCT05378893).

## 2 Materials and methods

### 2.1 Materials

ECL reading buffer, 96-well 1-Spot Sector plates (catalog number: L15XA-6), SULFO-TAG labeled Streptavidin (catalog number: R32AD-5), and the MESO QuickPlex SQ120 instrument were purchased from Meso Scale Diagnostics (Rockville, MD, USA). Tween-20 (catalog number: P1379) and bovine serum albumin (BSA, catalog number: B2064) were obtained from Sigma (Shanghai, China). 10 × PBS (catalog number: 5,460–0,023) was sourced from KPL (Milford, MA, USA). G418 (catalog number: ant-gn-5), hygromycin (catalog number: ant-hg-5), and puromycin (catalog number: ant-pr-1) were purchased from Invivogen (San Diego, CA, USA). Fetal bovine serum (FBS, catalog number: FSP500) was supplied by Excell Bio Company (Shanghai, China). DMEM/F12 (catalog number: L310KJ) and high glucose DMEM (catalog number: L110KJ) were sourced from BasalMedia (Shanghai, China). Goat Anti-Human IgG, Monkey ads-HRP (catalog number: 2049–05) and Goat Anti-Human IgG, Monkey ads-UNLB (catalog number: 2049–01) were obtained from SouthernBiotech (Birmingham, AL, USA).

### 2.2 Cell lines and culture conditions

Recombinant CHO-K1 GLP-1R Luc cells expressing the luciferase gene under the control of the cAMP response element (CRE) and human GLP-1R gene were cultured in DMEM/F12 medium supplemented with 10% FBS, 1 mg/mL G418, and 500 μg/mL hygromycin. Recombinant CHO-K1 GCGR Luc cells expressing CRE and human GCGR gene were cultured in DMEM/F12 medium supplemented with 10% FBS and 10 μg/mL puromycin. Recombinant HEK293 KLB Luc cells expressing a 5 × UAS-driven luciferase gene, KLB gene, and GAL4DBD ELK1 fusion protein were cultured in high-glucose DMEM supplemented with 10% FBS, 1 μg/mL puromycin, and 25 μg/mL hygromycin. All cells were maintained at 37 °C in a humidified incubator with 5% CO_2_.

### 2.3 *In vitro* cell potency assay

The CHO-K1 GLP-1R Luc or CHO-K1 GCGR Luc cell lines were used to assess the agonistic potency of DR10624 on GLP-1R or GCGR activation. Upon activation of these receptors, cAMP levels increase, which in turn activates the cAMP response elements (CREs) and induces luciferase gene expression. Cells were seeded at a density of 2.5 × 10^4^ cells per well in 96-well plates and incubated overnight. DR10624 and other test proteins were then added, and the plates were further incubated for 5 h. After incubation, the cells were lysed using the Promega Bright-Glo™ Luciferase Assay System (catalog number: E2620; Madison, WI, USA) and luciferase activity was measured using the Molecular Devices I3 microplate reader (San Jose, CA, USA).

The agonistic activity of DR10624 on the FGF21 receptor complex was evaluated using HEK293 KLB Luc cells. In this system, luciferase activity is regulated by the phosphorylation status of the fusion protein GAL4DBD-ELK1, which is induced upon binding of the FGF21 analog to the receptor complex. Cells were seeded at a density of 4 × 10^4^ cells per well in 96-well plates and incubated overnight. DR10624 and other test proteins were then added, and the plates were incubated for an additional 6 h. Afterward, the cells were lysed using the Bright-Glo™ Luciferase Assay System, and luciferase activity was measured using the I3 microplate reader.

### 2.4 Generation of DR10624 N- and C-terminal specific antibodies

The full-length N-terminal 29-amino acid hybrid peptide and the C-terminal 15-amino acid peptide of DR10624 (Supplementary Table S1), along with the N-terminal truncated peptide lacking two amino acids (ΔN2) and the C-terminal truncated peptide lacking three amino acids (ΔC3), were synthesized and conjugated to either Keyhole Limpet Hemocyanin (KLH) or BSA by QYAOBIO (Shanghai, China**).** For rabbit immunization, only the KLH-conjugated full-length peptides (29-mer for N-terminus and 15-mer for C-terminus) were used as immunogens. DR10624, DR10624 ΔN2, and DR10624 ΔC4 protein were prepared in-house and verified by SDS-PAGE, HPLC and LC-MS/MS (Supplementary Figure S1 and Supplementary Table S2).

Customized DR10624 N- and C-terminal specific antibodies were developed by Pyrogen Biosystems (Wuhan, China). Briefly, four healthy New Zealand white rabbits were immunized four times with KLH-conjugated full-length N-terminal or C-terminal peptides (exact same sequences as described above). The first immunization was administered with complete Freund’s adjuvant, followed by three subsequent immunizations with incomplete Freund’s adjuvant. Based on immune titer responses, one rabbit was selected and boosted a fifth time with the antigen. Peripheral blood mononuclear cells (PBMCs) were then isolated from the selected rabbit.

Antigen-specific B Cells were enriched using biotinylated DR10624-conjugated magnetic beads and seeded into twenty 96-well plates using the limited-dilution method. Culture supernatants were screened by ELISA (Enzyme-linked immunosorbent assay), and positive clones that specifically bound to DR10624 or BSA conjugated full length N- or C- terminalpeptide, but not to BSA-conjugated ΔN2 or ΔC3 peptides, were selected. RNA was extracted from these clones, and reverse-transcribed cDNA was sequenced and cloned into an expression vector. The resulting constructs were transiently expressed in HEK293F cells, and the antibodies were purified using protein A resin according to the manufacturer’s instructions (NanoMicro, catalog number: 17013–070100; Suzhou, China). Antibody specificity was further confirmed by ELISA.

### 2.5 Sodium dodecyl sulfate polyacrylamide gel electrophoresis (SDS-PAGE)

Protein samples (as indicated) were mixed with sample loading buffer, heated at 70 °C for 10 min, and quickly spun down. SDS–PAGE was performed using 5% stacking and 10% resolving gels on a vertical electrophoresis system (Tanon, Shanghai, China). Electrophoresis was run at 85 V through the stacking gel and 150 V through the resolving gel. Gels were stained and destained, and images were acquired on a ChemiScope 6,100 imaging system (CLINX, Shanghai, China).

### 2.6 Size exclusion high-performance liquid chromatography (SEC-HPLC)

SEC-HPLC was performed on an Agilent 1110 HPLC system (Santa Clara, CA, USA) equipped with a Superdex 200 Increase 5/150 GL column (Cytiva, Marlborough, MA, USA). Samples were run isocratically at 0.2 mL/min in 150 mM phosphate buffer (pH 7.0) containing 5% (v/v) isopropanol (Merck, catalog number: 101040, St. Louis, MO, USA) as the mobile phase, and protein elution was monitored by UV absorbance at 214 nm. The column and autosampler were maintained at 35 °C and 10 °C, respectively.

### 2.7 Terminal sequencing of DR10624 and its truncation mutant

N- and C-terminal sequencing of DR10624 and its truncation mutants were conducted by GenScript (Nanjing, China). Briefly, 100 ug of protein was denatured and reduced with guanidine hydrochloride and DTT, followed by alkylation with iodoacetamide. After buffer exchange into ammonium bicarbonate, the protein was digested with trypsin or chymotrypsin for 4 h at 37 °C, and the reaction was quenched with formic acid. Accurate-mass data were acquired on a Waters (Milford, MA, USA) UPLC equipped with an ACQUITY UPLC Protein BEH C18 column and coupled to a Thermo Q Exactive mass spectrometer (Waltham, MA, USA).

### 2.8 Cell-based neutralization assays

Cell-based assays were conducted to evaluate the neutralizing capacity of the isolated N-terminal and C-terminal antibodies against DR10624. Briefly, CHO-K1 GLP-1R Luc, CHO-K1 GCGR Luc, and HEK293 KLB Luc reporter cells were seeded into 96-well plates and incubated overnight. A fixed concentration of DR10624 was pre-incubated with a serial dilution of each antibody for 1 h at room temperature before being added to the cell culture plates. Following incubation for the indicated time period, the cells were lysed using the Bright-Glo™ Luciferase Assay System, and luciferase activity was measured using the I3 microplate reader.

### 2.9 Pharmacokinetic comparison with different assays

Twelve cynomolgus monkeys (six females and six males) were administered 3 mg/kg of DR10624 subcutaneously. Blood samples were collected prior to administration, and at 2, 6, 24, 48, 72, 96, 120, and 168 h post-administration. Approximately 0.6 mL whole blood was collected into 2 mL tubes (catalog number: KJ020AS; Kangjian Medical, Taizhou, China) containing gel separator and coated with micronized silica as clot activator. Tubes were kept on ice for 2 h to facilitate clotting, then centrifuged at 2,000 × g for 10 min. The serum was transferred to Eppendorf tubes and stored at −80 °C for long-term preservation. Serum concentrations of DR10624 were measured using the ELISA method with two pairs of antibodies. The total concentration of DR10624 was detected using the goat anti-human IgG polyclonal antibodies in combination with the HRP labeled goat anti-human IgG polyclonal antibodies. To detect the concentration of intact DR10624, a pair of the antibodies specific to the N-terminus (clone 4G7) and C-terminus (clone 1A12) was used.

To evaluate the residual bioactivity of DR10624 *in vivo*, serum samples collected at 24, 96, and 168 h post-administration were diluted to equal concentrations based on the values determined by the total drug assay. These normalized samples were then analyzed for their agonistic potencies against GLP-1R, GCGR, and FGF21R following the cells-based assay procedures described in Section 2.3. Animal welfare and experimental procedures are in accordance with the provision of the Animal Ethics Committee of Shanghai InnoStar Bio-tech Co., Ltd. (Approval number: IACUC-2019-M-046).

### 2.10 ECL based method development

To improve the sensitivity of the method, an electrochemiluminescence (ECL)-based immunoassay was developed for the quantification of DR10624 in clinical trial samples.

MSD 96-well plates were coated with 50 μL of 250 ng/mL DR10624 N-terminal antibody (clone 4G7) at 4 °C for 14–24 h. Following the coating step, plates were washed and then blocked with 150 μL of 3% BSA in washing buffer for at least 1 hour. DR10624 standards and quality control (QC) samples were prepared in healthy human serum and diluted at a minimum required dilution (MRD) of 1:50 using assay buffer containing 1% BSA. After washing, 50 μL of diluted standards and samples were added to the plate and incubated for approximately 2 h at room temperature. Subsequently, plates were washed, and 50 μL of 500 ng/mL biotin-labeled DR10624 C-terminal antibody (clone 1A12) was added and incubated for about 1 hour. After another washing step, 50 μL of 100 ng/mL SULFO-TAG–labeled streptavidin was added to each well and incubated for an additional 30 min. Following a final wash, MSD Gold Read Buffer was added, and ECL signals were immediately measured using the MSD QuickPlex SQ120 instrument. The standard curve was fitted using a four-parameter logistic (4 PL) regression model with 1/Y^2^ weighting (Supplementary Table S3). The signal produced is proportional to the amount of analyte present and interpolated from the calibration curve present on each plate.

### 2.11 Method validation

The ECL-based method for quantifying DR10624 concentrations in human serum was validated in accordance with regulatory guidance (ICH M10 (Guideline, 2022), Chinese pharmacopoeia (Commission, 2020) and FDA Bioanalytical Method Validation Guidance for Industry (USFaD, Administration, 2018)).

### 2.12 Statistical analysis

All statistical analyses were performed using GraphPad Prism version 10.1.2 (GraphPad Software Inc., Boston, MA, USA) or Microsoft Excel, Microsoft Office LTSC Professional plus 2024. PK parameters were calculated with an Excel add-in software PKsolver (Zhang et al., 2010). The paired *t*-test was used to calculate the difference and a *p-*value of < 0.05 was considered to be significant.

## 3 Results and discussions

### 3.1 The relationship between DR10624 bioactivity and N/C terminal integrity

**FIGURE 1:**
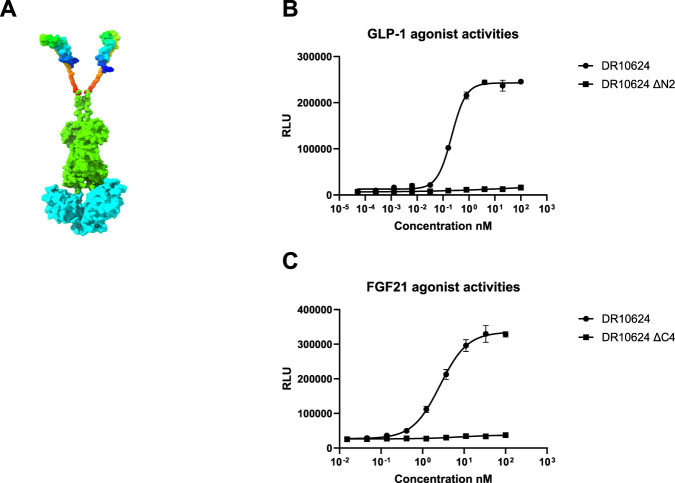
The importance of N-terminus and C-terminus for DR10624. **(A)** Schematic of DR10624 structure. The GLP-1 and GCG dual agonist peptide (mixed color) was linked to the N terminus of IgG1 Fc (Green) with 3 × G_4_S linker (orange), and FGF21 (indigo blue) was fused to the C terminus with 3 × G_4_A linker. **(B)** GLP-1R agonist activities of full DR10624 and N terminal two amino acids truncated DR10624 ΔN2. CHO-K1 GLP-1R Luc cells were seeded and incubated overnight. DR10624 and DR10624 ΔN2 were added at a starting concentration of 100 nM followed by five-fold serial dilutions. After a further 5 h incubation, luciferase activity was measured. **(C)** FGF21R agonist activities of full DR10624 and C terminal four amino acids truncated DR10624 ΔC4. HEK293 KLB Luc cells were seeded and incubated overnight. DR10624 and DR10624 ΔC4 were added to the cells at a starting concentration of 100 nM and serially diluted threefold. After a further 6 h incubation, luciferase activity was measured.

DR10624 is a human IgG1 Fc (N297A) fusion protein designed for half-life extension, with a theoretical molecular weight of approximately 101.8 kDa. Structurally, the dual functional GLP-1/GCG hybrid peptide is linked to the N-terminus through a flexible 3 × G_4_S linker, while the mutated human FGF21 moiety is fused to the C-terminus of the Fc region via a flexible 3 × G_4_A linker (Figure 1A). Given that the intact N-terminus is critical for the incretin agonist activity and the C-terminus plays a vital role in FGF21 activity, we conducted assays to evaluate the specific roles of the N- and C-termini of DR10624.

As shown in Figure 1B, compared to the full-length DR10624, the removal of the two N-terminal amino acids (His and Ser) in DR10624 (ΔN2) completely abolished its GLP-1R agonist activity, highlighting the critical role of these amino acids in DR10624’s function. In previous report (Micanovic et al., 2009), Radmila Micanovic et al. examined the role of the FGF21 C-terminus in MAPK activation by measuring phospho-ERK1/2, and found that FGF21 ΔC4 retained with only ∼6% relative potency compared with FGF21 wild-type. While in our study, removal of the C-terminus to create DR10624 ΔC4 (Ser, Tyr, Glu, and Ser) rendered the truncation completely inactive, even at the highest concentration, which was unexpected (Figure 1C).

The observed differences between our results and previous findings may arise from methodological variations in the assay systems. In the current study, we employed a phospho-ELK1-driven cell-based luciferase reporter system (a downstream component of the phospho-ERK1/2 signaling cascade). Meanwhile, the absolute loss of activity in DR10624 ΔC4 highlights a unique structural dependency. Unlike wild-type FGF21, DR10624 features five amino acid substitutions engineered to optimize therapeutic properties. These substitutions may structurally cross-talk with the C-terminal tail, meaning that the ΔC4 truncation completely destabilizes the co-receptor β-Klotho binding pocket. This structural disruption, combined with the stringent transcription thresholds of our downstream phospho-ELK1-driven luciferase assay, accounts for the complete lack of detectable agonism compared to the weak residual signals historically reported for wild-type truncations. Collectively, the results indicate that both the N- and C-termini of DR10624 are critical for its biological functions.

### 3.2 DR10624 N- and C-terminal specific antibodies

Given the importance of structural integrity for DR10624’s biological activity, we aim to develop specific antibodies targeting both the N- and C-termini, particularly the last two or three amino acids. New Zealand rabbits were immunized with KLH-conjugated full-length N-terminal or C-terminal peptides, respectively. Single B Cells producing culture supernatants that bound specifically to DR10624—but not to BSA-conjugated truncated peptides or a human IgG mixture—were selected for sequencing (Flow chart shown in Supplementary Figure S2). The corresponding antibodies were then expressed, purified and characterized (Supplementary Figure S3).

**FIGURE 2:**
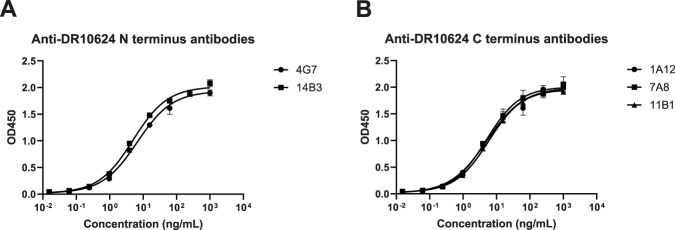
The binding activities of N terminal and C terminal antibodies. **(A)** The binding activities of N terminal antibodies 4G7 and 14B3 to DR10624. **(B)** The binding activities of C terminal antibodies 1A12, 7A8 and 11B1 to DR10624. One hundred nanograms per well of DR10624 were coated onto an ELISA plate overnight and the wells were blocked. Antibodies were added at the indicated concentrations and incubated for 2 h, followed by washing. HRP-labeled goat anti-rabbit polyclonal antibodies were incubated for 1 h, washed, and TMB substrate was added, incubated for 5 min at room temperature and stopped with 2 M sulfuric acid. Absorbance was measured at 450 nm.

For the N-terminus, 387 clones were found to bind DR10624, of which 18 did not bind to BSA-conjugated ΔN2. Among them, only clones 4G7, 5A12, 7F12, and 14B3 were successfully sequenced and expressed (Supplementary Table S4). In subsequent characterization, 5A12 and 7F12 lost their binding activity (Supplementary Table S5). In contrast, clones 4G7 and 14B3 specifically bound to DR10624 (Figure 2A), showed reactivity to BSA-conjugated ΔN2 only at high concentrations, and did not bind to human IgG (Supplementary Figures S4A, S4B).

For the C-terminus, only 26 clones showed binding to DR10624, 15 of which did not bind to BSA-conjugated ΔC3 or human IgG (Supplementary Table S4). Of the 15 clones successfully expressed, 14 bound to DR10624 but not to BSA-conjugated ΔC3 (Supplementary Table S5). As all the 14 clones met our prespecified criteria, we only selected clones 1A12, 7A8, and 11B1 for further expansion in HEK293F cells. All three specifically bound to DR10624 (Figure 2B) and exhibited no binding to BSA-conjugated ΔC3 or human IgG (Supplementary Figures S4C, 4D).

### 3.3 Antibodies bind to the active domain of DR10624

**FIGURE 3:**
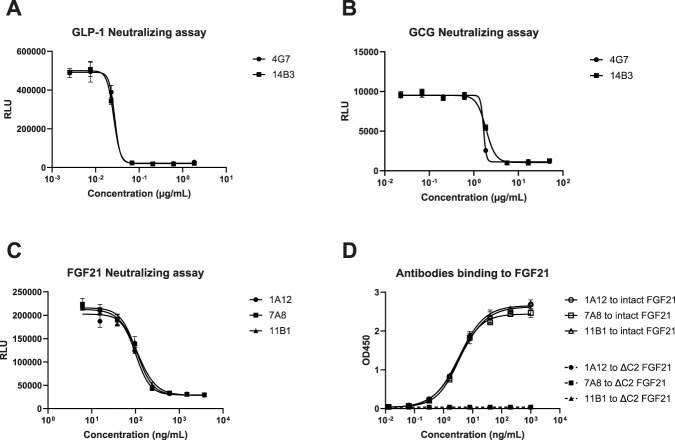
The neutralizing activities of the N- and C- terminal antibodies. **(A)** GLP-1 neutralizing activities of 4G7 and 14B3 to DR10624. CHO-K1 GLP-1R Luc cells were seeded and incubated overnight. 0.6 nM DR10624 and gradient-diluted antibodies were pre-incubated for 1 h and added to the cells, and then incubated for 5 h, then the luciferase activity was measured. **(B)** GCG neutralizing activities of 4G7 and 14B3 to DR10624. CHO-K1 GCGR Luc cells were seeded and incubated overnight. 14 nM DR10624 and gradient-diluted antibodies were pre-incubated for 1 h and added to the cells, and then incubated for 5 h, then the luciferase activity was measured. **(C)** FGF21 neutralizing activities of 1A12, 7A8 and 11B1 to DR10624. HEK293 KLB Luc cells were seeded and incubated overnight. 8.0 nM DR10624 and gradient-diluted antibodies were pre-incubated for 1 h and added to the cells, and then incubated for 6 h, then the luciferase activity was measured. **(D)** Binding specificities of the C terminal antibody 1A12, 7A8 and 11B1 to FGF21. One hundred nanograms per well of FGF21 or ΔC2 were coated onto an ELISA plate overnight and the wells were blocked. Antibodies were added at the indicated concentrations and incubated for 2 h, followed by washing. HRP labeled goat anti rabbit polyclonal antibodies were incubated for 1 h, washed, and TMB substrate was added, incubated for 5 min and stopped with 2 M sulfuric acid. Absorbance was measured at 450 nm.

The binding capacities of the antibodies to the active domains were evaluated via a cell potency assay. As shown in Figures 3A, B, the N-terminal antibodies, clones 4G7 and 14B3, neutralized both the GLP-1R and GCGR agonist activities of DR10624. Similarly, the C-terminal antibodies, clones 1A12, 7A8 and 11B1, blocked the FGF21R agonist activity of DR10624 (Figure 3C). These results confirmed that all the isolated antibodies bind to the active domain of DR10624.

Since the C-terminal integrity of FGF21 is critical for its binding to KLB and the C-terminal two amino acids truncation led to about 90% loss of potency (Yie et al., 2009), we further verified whether 1A12, 7A8 and 11B1 bind to this key site. Full-length FGF21, along with FGF21 ΔC2 truncation derived from DR10624, were used as capture antigens. As shown in Figure 3D, the ΔC2 truncation showed no binding affinity to either 1A12, 7A8 or 11B1. This indicates that the C terminal two amino acid residues of the FGF21 from DR10624 is a critical binding epitope for 1A12, 7A8 and 11B1.

### 3.4 Development of a sandwich ELISA for DR10624 quantification

The sandwich ELISA is widely regarded as a sensitive and specific method for the rapid and accurate quantification of protein therapeutics in clinical trials (Fang et al., 2024; Lv et al., 2019; Yoshida et al., 2012). To identify the optimal antibody pair for DR10624 detection, antibodies targeting the N- and C-termini were evaluated in a sandwich ELISA format.

N-terminal or C-terminal antibodies were used as capture antibodies, while the complementary C-terminal or N-terminal antibodies were employed as detection antibodies. All possible pairings were tested by titrating DR10624 and assessing the response.

**FIGURE 4:**
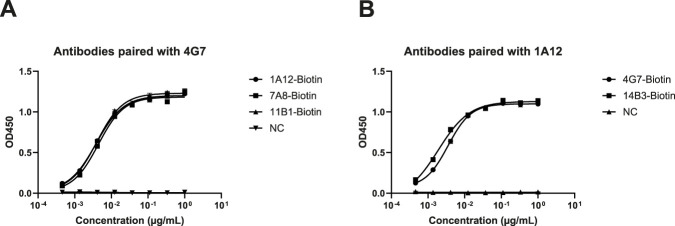
Antibody pairs for the quantification of DR10624. **(A)** Antibody pairs with 4G7 coated as capture antibody and biotinylated 1A12, 7A8 and 11B1 as detection antibody, respectively. **(B)** Antibody pairs with 1A12 coated as capture antibody and biotinylated 4G7, 14B3 as detection antibody, respectively. Capture antibody (200 ng/well) was coated onto an ELISA plate overnight, and the wells were blocked. DR10624 was added at a starting concentration of 1,000 ng/mL and serially diluted threefold; plates were incubated for 1 h and washed. Biotin-labeled detection antibody (1,000 ng/mL) was added, incubated for 1 h, and washed. Streptavidin-HRP (1:10,000) was added and incubated for 1 h. TMB substrate was then added and incubated for 5 min, after which the reaction was stopped and absorbance was measured at 450 nm.

As shown in Figure 4 and Supplementary Figure S5, all antibody combinations produced comparable titer curves, regardless of which antibody was used for capture or detection. Since no significant differences were observed among the pairs in initial binding titers, the final pairing configuration was rigorously determined through a systematic assessment of biophysical developability, stability, and assay performance. For the N-terminus, clone 4G7 was selected as the capture antibody due to its excellent monomeric purity (98.1% via SEC-HPLC) and structural stability, whereas clone 14B3 was excluded because it exhibited poor long-term storage stability, with its monomeric purity dropping drastically to 56.1% upon prolonged storage. For the C-terminus, clone 1A12 was selected as the biotinylated detection reagent because the 1A12-Biotin configuration achieved higher accuracy in buffer QCs and yielded robust, acceptable recoveries for both high and low QCs across individual human serum matrices (data not shown), ensuring high data reliability for ongoing clinical sample analysis.

### 3.5 The influence of assay formats on PK parameters

The area under the curve (AUC) and maximum concentration (C_max_) are key pharmacokinetic (PK) parameters commonly used to evaluate a drug’s systemic exposure. To assess how assay methodology impacts the measurement of drug exposure, the PK profile of DR10624 was examined in non-human primates.

Twelve cynomolgus monkeys were administered subcutaneously a single 3 mg/kg dose of DR10624, and blood samples were collected at specified time points. Two distinct ELISA configurations were used for quantification: a combination of the monkey serum pre-absorbed goat anti human Fc polyclonal antibodies employed as both capture and detection reagents to measure total DR10624, and the 4G7/1A12 antibody pair to quantify intact DR10624.

**FIGURE 5:**
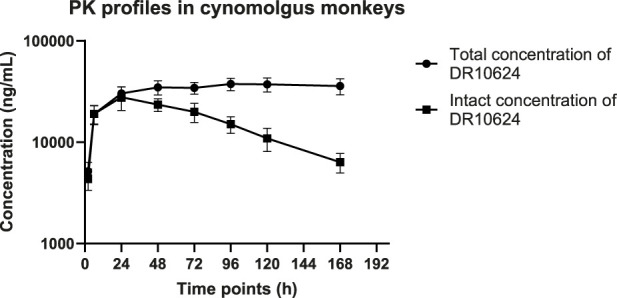
PK profiles of DR10624 in cynomolgus monkeys. Twelve cynomolgus monkeys (six females and six males) received 3 mg/kg DR10624 by subcutaneous injection, and blood samples were collected at the indicated time points. Total DR10624 concentrations were measured using monkey serum–preabsorbed goat anti-human IgG polyclonal antibodies as both capture and detection reagents, while intact DR10624 was quantified with the 4G7 and 1A12 antibody pair.

As demonstrated in Figure 5, the concentrations of DR10624 in the total format were higher than those in the intact format across the entire sampling period, particularly after the 48 h time point. Furthermore, the systemic exposure of DR10624 measured by the intact format was observed to be significantly lower than that measured by the total format (Table 1). These results suggest that DR10624 may be unstable *in vivo* and highlight the importance of assay selection for accurately assessing DR10624 exposure in clinical trials.

**TABLE 1:** PK parameters of DR10624 in cynomolgus monkeys (Mean ± SD).

PK parameters	Unit	Intact DR10624	Total DR10624	*p*-value^1^
		n = 12	n = 12	
C_max_	μg/mL	28.0 ± 7.0	39.4 ± 6.0	0.0005
AUC _0–168h_	h*μg/mL	2,753 ± 490	5,616 ± 759	<0.0001
AUC _inf_	h*μg/mL	3,330 ± 598	NA^2^	​
AUC_extra_	%	17% ± 5%	NA	​
CL/F	mL/h/kg	0.9 ± 0.2	NA	​
V_z_/F	mL/kg	80 ± 16	NA	​
T_max_	h	32 ± 12	108 ± 33	​
*t*_1/2_	h	61 ± 14	NA	​

1 *p*-value was calculated from paired *t*-test between intact and total method, and the normality of the difference was test with use of Shapiro-Wilk test (C_max_ *p*-value = 0.9824, AUC *p*-value = 0.7485).

2 Not Applicable due to the absence of an observed elimination phase.

Beyond *in vivo* degradation of DR10624, methodological differences between the two assay configurations may partially contribute to the observed exposure discrepancy. The total format assay utilizes polyclonal antibodies directed against the Fc region, offering high binding avidity that is generally resilient to local structural changes. In contrast, the intact format assay relies on a strict dual-monoclonal configuration targeting specific terminal epitopes. Consequently, the intact assay is inherently more sensitive to potential assay-related artifacts, such as target-mediated receptor interference or epitope masking by endogenous serum proteins, which can obstruct monoclonal binding and contribute to the lower circulating concentrations of intact DR10624.

To evaluate the *in vivo* stability of DR10624, intact mass analysis was performed using LC-MS on serum samples collected from cynomolgus monkeys at the 168-h time point. As shown in Supplementary Figure S6 and Supplementary Table S6, four truncation fragments were identified, corresponding to cleavage sites between Pro31-Ser32, Gly448-Ile449, and Ser475-Tyr476. These truncations likely contribute to the observed reduction in intact drug exposure. Additionally, other potential mechanisms—such as epitope masking or complex formation—may further explain the lower circulating concentrations of intact DR10624.

To further verify the functional viability of the circulating drug over time, the agonistic activities of serum samples collected at 24 h, 96 h, and 168 h post-administration in cynomolgus monkeys were evaluated using cell-based luciferase reporter assays. The results revealed that the agonistic potencies on both GLP-1R and GCGR decreased dramatically from 24 h to 168 h (Supplementary Figure S7). Conversely, the agonistic activity on FGF21R demonstrated only a mild decline over the same time course. Specifically, the rapid drop in GLP-1R and GCGR activities, compared to the milder decline in FGF21R activity, hinted that the N-terminus of DR10624 may encounter a tighter proteolytic bottleneck in circulation than its C-terminus. By bridging antibody-based concentration analysis with cell-based functional results, we are able to confirm that terminus-intact assay essentially reflects pharmacologically active molecules *in vivo*, highlighting the practical value of using a dual-lock sandwich configuration for multi-specific drug characterization.

### 3.6 ECL based dual-monoclonal sandwich immunoassay method validation

Given that the initial dose of DR10624 in the Phase I clinical study conducted in healthy adults was 0.5 mg per person, administered subcutaneously, the serum concentrations in the first cohort were expected to be quite low. Furthermore, the MSD ECL platform is widely recognized for its advantages in terms of low background interference and high sensitivity (Wu et al., 2013). To gain a better understanding of the pharmacokinetic (PK) profiles of DR10624 in the human body, we chose to develop and validate an ECL-based dual-monoclonal sandwich immunoassay for determining its concentration. The N-terminal antibody clone 4G7 was used as the capture antibody, paired with the biotin-labeled C-terminal antibody clone 1A12 as detection antibody.

**FIGURE 6:**
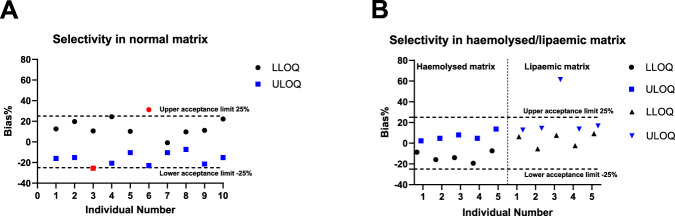
Results for selectivity **(A)**. Selectivity at LLOQ and ULOQ in healthy human serum matrices. Ten healthy human serum samples were spiked with DR10624 at the LLOQ (5.00 ng/mL) and ULOQ (1,250 ng/mL). Back-calculated recoveries were then compared against the corresponding theoretical concentrations. **(B)** Selectivity at LLOQ and ULOQ in haemolysed or lipaemic matrices. Five haemolysed or lipaemic serum samples were spiked with DR10624 at the LLOQ (5.00 ng/mL) and ULOQ (1,250 ng/mL). Back-calculated recoveries were then compared against the corresponding theoretical concentrations. LLOQ: Lower limit of quantification; ULOQ: Upper limit of quantification.

The ECL-based immunoassay successfully detected DR10624 across a wide concentration range in serum (5.00–1,250 ng/mL), with a representative standard curve shown in Supplementary Figure S8. Prior to analysis, samples were subjected to a minimum required dilution (MRD) of 1:50. The method was fully validated in accordance with regulatory guidelines, and a summary of the validation results is provided in Table 2. Owing to its excellent accuracy and precision, the assay enabled reliable quantification of DR10624 in human serum, with a sensitivity of 5.00 ng/mL. Furthermore, selectivity testing in normal, hemolyzed, and lipaemic samples were conducted, and all results complied with ICH M10 guidelines. While one lipemic ULOQ sample showed deviations (56.0% CV and 61.6% RE) from acceptance criteria, potentially due to procedural variability, blank and LLOQ lipemic samples (n = 5 each) consistently met validation standards (CV ≤ 20%, RE within ±20%). This confirmation supports the reliability of the ECL method for quantifying DR10624 in Phase II clinical trials involving HTG patients (Figure 6 and Supplementary Table S7). No hook effect was observed up to 200,000 ng/mL, a level far exceeding clinically relevant concentrations. In addition, robust dilutional linearity with the dilution factor of up to 4,000 supported accurate quantification of samples above the ULOQ (Supplementary Table S8). This validation was particularly relevant given the highest anticipated clinical dose (∼120 mg per subject), which theoretically yields a maximum concentration of ∼24 μg/mL (assuming a blood volume of 5.0 L), and the dilution linearity of up to 1:4,000 ensures accurate quantitation even at peak exposure levels. Meanwhile, DR10624 remained stable when stored at 2 °C–8 °C and at room temperature for up to 24 h, and at −80 °C for 195 days, as well as after six repeated freeze–thaw cycles, supporting its routine quantification in human serum matrices.

**TABLE 2:** The overall summary table for the ECL method validation (ng/mL).

Matrix	healthy human serum				
MRD	1:50				
Calibration curve range	5.00–1,250				
Accuracy and Precision (N = 35)^1^	LLOQ	LQC	MQC	HQC	ULOQ
	5.00	15.0	100	950	1,250
Accuracy (RE%)	9.2	9.3	5.0	14.7	−4.8
Precision (CV%)	11.1	9.6	9.4	10.5	13.9
Total error %	20.3	18.9	14.4	25.2	18.7
Selectivity	Blank	​	LLOQ	​	ULOQ
Normal serum matrix (N = 10)	10/10	​	9/10	​	9/10
Haemolysed matrix (N = 5)	5/5	​	5/5	​	5/5
Lipaemic matrix (N = 5)	5/5	​	5/5	​	4/5
Dilutional linearity and hook effect					
Dilutional linearity	Up to 1:4,000 diluted in serum matrix				
Hook effect	No hook effect observed up to 200,000 ng/mL				
Stabilities					
Freeze-thaw stability	Stored at nominal −80 °C and thawed at RT: 6 cycles				
Short-term stability	Up to 24 h 17 min at 2 °C–8 °C and RT				
Long-term stability	Stored at nominal −80 °C: 195 days				

1 LLOQ, lower limit of quantification; LQC, low quality control; MQC, medium quality control; HQC, high quality control; ULOQ, upper limit of quantification; RE, relative error; CV, coefficient of variation.

To rigorously address analytical specificity within complex clinical serum matrices, cross-reactivity against endogenous human hormone counterparts was systematically mapped via ELISA (Supplementary Figure S9). The N-terminal clone 4G7 bound specifically to the engineered N-terminus of DR10624 and exhibited cross-reactivity with endogenous human GCG due to sequence homology, but showed no binding toward endogenous GLP-1 or FGF21. Conversely, the C-terminal clone 1A12 bound to endogenous human FGF21 due to sequence conservation at the extreme C-terminus, but exhibited no cross-reactivity with endogenous GLP-1 or GCG.

Crucially, the dual-monoclonal sandwich configuration (4G7 as capture and 1A12 as detection) functioned as a strict dual-lock gating system that avoided any potential single-domain cross-reactivity. Endogenous human GCG molecules captured by 4G7 could not be bound by 1A12, whereas endogenous human FGF21 could not be captured by 4G7 in the initial step and was washed away. Furthermore, selectivity test results for LLOQ levels of DR10624 in human serum matrices containing physiological baseline levels of these hormones consistently complied with ICH M10 guidelines. Meanwhile, peak circulating endogenous GCG concentration (∼ 103 pg/mL in diabetes patients) is fundamentally negligible compared to the much higher expected therapeutic serum concentrations of DR10624 in patients (Cooperberg and Cryer, 2009). This confirms that the assay achieves absolute analytical specificity and is entirely resilient to endogenous hormone fluctuations, fully supporting its application in Phase II clinical trials involving patients with metabolic disorders.

Overall, the ECL-based immunoassay fulfilled the regulatory criteria (Supplementary Table S9) and demonstrated suitability for the quantification of active DR10624 in clinical studies.

## 4 Conclusion

DR10624 is a tri-agonist Fc-fusion protein that combines GLP-1/GCG activity at the N-terminus with FGF21 at the C-terminus. Structure–function data confirmed that minimal truncations at either terminus of DR10624 abolish corresponding agonism, motivating an assay strategy that distinguishes intact, bioactive drug from degraded forms. We generated terminus-specific neutralizing monoclonal antibodies and established a sandwich immunoassay using 4G7 for N-terminal capture and biotinylated 1A12 for C-terminal detection to selectively quantify intact DR10624. In cynomolgus monkeys, intact concentrations were notably lower than total DR10624, indicating *in vivo* instability and emphasizing the need for integrity-selective measurements in PK assessment. To cover the pharmacokinetic analysis of low dosage of DR10624 in clinical trial, a sensitive ECL-based immunoassay was established and validated. The assay met regulatory expectations for accuracy, precision, selectivity, dilution linearity and hook effect, and stability across a 5.00–1,250 ng/mL range. To guarantee robust performance for upcoming Phase II trials in hypertriglyceridemia patients as one lipemic ULOQ sample failed to meet the acceptance criteria, our clinical sample analysis protocol mandates that all study samples be analyzed in duplicate, and any sample showing an intra-plate CV > 20% will trigger re-analysis according to pre-defined clinical testing SOPs.

Collectively, this robust, sensitive, and specific method enables reliable monitoring of bioactive DR10624 exposure, supporting clinical development in SHTG and related disorders, and providing a generalizable framework for integrity-based bioanalysis of multi-specific protein therapeutics.

## Data Availability

The original contributions presented in the study are included in the article/Supplementary Material, further inquiries can be directed to the corresponding authors.
