# Supplementary material for: Terminus-specific antibody development and bioanalytical assay validation to quantify active DR10624 in pharmacokinetic studies
Source: Front Pharmacol. 2026 Aug 3;17:1863402. doi: 10.3389/fphar.2026.1863402 (PMC13477871; doi:10.3389/fphar.2026.1863402)
Supplement: Supplementary file 1 [file Supplementaryfile1.docx]

**Terminus-specific antibody development and bioanalytical assay validation to quantify active DR10624 in pharmacokinetic studies**

Zhenxing Zhou^1^, Kexin Cao^1^, Gaofeng Yao^1^, Yuanyuan Liu^1^, Wenwen Duan^1^, Chengcheng Yang^1^, Shimei Sheng^1^, Yanshan Huang^1,*^

^1^ Department of Innovative Drug Discovery and Development, Zhejiang Doer Biologics Co., Ltd., Hangzhou, China

^*^Corresponding author.

E-mail address: yanshanhuang@doerbio.com (Y. Huang)

**Table S1** Peptide sequences used in this study

| Name | Sequence |
| --- | --- |
| N-terminal peptide | **HS**QGTFTSDYSKYLDSQAAQDFVQWLLAG |
| KLH Conjugated N terminal peptide | **HS**QGTFTSDYSKYLDSQAAQDFVQWLLAGC-KLH |
| BSA Conjugated N terminal peptide | **HS**QGTFTSDYSKYLDSQAAQDFVQWLLAGC-BSA |
| BSA-ΔN2 | QGTFTSDYSKYLDSQAAQDFVQWLLAGC-BSA |
| C-terminal peptide | SMVGGSQGRSPS**YES** |
| KLH Conjugated C terminal peptide | KLH-CSMVGGSQGRSPS**YES** |
| BSA Conjugated C terminal peptide | BSA-CSMVGGSQGRSPS**YES** |
| BSA-ΔC3 | BSA-CSMVGGSQGRSPS |

**Table S2** Terminal sequencing of DR10624 and its terminal truncation mutants by LC-MS/MS

| Samples | C terminal sequence | N terminal sequence | Experimental  Mass | Theoretical Mass | ∆ ppm |
| --- | --- | --- | --- | --- | --- |
| DR10624 | SPSYES | — | 668.2659 | 668.2653 | 0.90 |
| DR10624ΔC4 | SMVGGSQGRSP | — | 1061.4951 | 1061.4924 | 2.54 |
| DR10624 | — | HSQGTFTSDYSK | 1356.5992 | 1356.5946 | 3.39 |
| DR10624ΔN2 | — | QGTFTSDYSK | 1115.479 | 1115.4771 | 1.70 |

**Table S3** Data acquisition equipment and parameters

| Plate Type | MSD Standard Bind Plate |
| --- | --- |
| Plate Reader | MSD S120 Quickplex |
| Data collection | MSD Methodcial Mind V1.0.38 |
| LM1 | N/A |
| LM2 | N/A |
| Shake | N/A |
| Blank Subtraction | None |
| Curve Fit | 4-PL**^1^** |
| Weighting | 1/y2 |
| Units | ng/ml |

**^1^** Y=(A-D)/(1+(X/C)^B^)+D, where A and D represents the minimum and maximum asymptotes, respectively. C represents the curve midpoint and B represents the curve slope factor.

**Table S4** Binding specificities of isolated B clones culture supernatants (OD450)

| N terminal antibody | | | | | C terminal antibody | | | |
| --- | --- | --- | --- | --- | --- | --- | --- | --- |
| clone | DR10624 | Intact N peptide | ΔN2 peptide | human IgG | clone | DR10624 | ΔC3 peptide | human IgG |
| **4G7** | 1.025 | 1.076 | 0.109 | 0.017 | **1A12** | 1.050 | 0.034 | 0.034 |
| 5A12 | 0.985 | 0.627 | 0.026 | 0.088 | 1B11 | 1.074 | 0.068 | 0.133 |
| 7F12 | 0.802 | 0.869 | 0.023 | 0.021 | 2G4 | 1.030 | 0.014 | 0.140 |
| **14B3** | 0.944 | 1.040 | 0.153 | 0.009 | 2G9 | 1.118 | 0.091 | 0.098 |
|  |  |  |  |  | 3E5 | 1.014 | 0.030 | 0.094 |
|  |  |  |  |  | 3H4 | 0.910 | 0.018 | 0.140 |
|  |  |  |  |  | 6B8 | 1.120 | 0.076 | 0.107 |
|  |  |  |  |  | **7A8** | 1.040 | 0.029 | 0.144 |
|  |  |  |  |  | 10E10 | 1.110 | 0.034 | 0.181 |
|  |  |  |  |  | 10F6 | 0.815 | 0.035 | 0.030 |
|  |  |  |  |  | **11B1** | 1.105 | 0.020 | 0.123 |
|  |  |  |  |  | 11C1 | 1.078 | 0.009 | 0.164 |
|  |  |  |  |  | 11D12 | 1.090 | 0.019 | 0.147 |
|  |  |  |  |  | 11E9 | 1.072 | 0.026 | 0.103 |
|  |  |  |  |  | 12G11 | 0.983 | 0.027 | 0.116 |

**Table S5** Binding specificities of recombinant expressed products (OD450)

| Dilution | N terminal antibody | | | | | | | |
| --- | --- | --- | --- | --- | --- | --- | --- | --- |
|  | ΔN2 peptide | | | | DR10624 | | | |
|  | **4G7** | 5A12 | 7F12 | **14B3** | **4G7** | 5A12 | 7F12 | **14B3** |
| 1:2 | 0.383 | 0.015 | 0.02 | 0.304 | 1.337 | 0.062 | 0.042 | 1.316 |
| 1:6 | 0.292 | 0.006 | 0.005 | 0.227 | 1.254 | 0.02 | 0.023 | 1.283 |
| 1:18 | 0.197 | 0.014 | 0.014 | 0.143 | 1.209 | 0.024 | 0.015 | 1.192 |
| 1:54 | 0.105 | 0.009 | 0.003 | 0.068 | 1.124 | 0.016 | 0.011 | 1.12 |
| Dilution | C terminal antibody | | | | | | | |
|  | ΔC3 peptide | | | | DR10624 | | | |
|  | **1A12** | 1B11 | 2G4 | 2G9 | **1A12** | 1B11 | 2G4 | 2G9 |
| 1:2 | 0.028 | 0.084 | 0.035 | 0.095 | 1.085 | 0.996 | 1.02 | 1.056 |
| 1:6 | 0.018 | 0.055 | 0.033 | 0.071 | 1.054 | 1.062 | 0.98 | 1.098 |
| 1:18 | 0.011 | 0.051 | 0.029 | 0.061 | 1.034 | 0.983 | 0.943 | 1.023 |
| 1:54 | 0.011 | 0.029 | 0.022 | 0.049 | 0.989 | 0.921 | 0.861 | 1.001 |
| Dilution | C terminal antibody | | | | | | | |
|  | ΔC3 peptide | | | | DR10624 | | | |
|  | 3E5 | 3H4 | 6B8 | **7A8** | 3E5 | 3H4 | 6B8 | **7A8** |
| 1:2 | 0.098 | 0.092 | 0.05 | 0.015 | 1.088 | 1.108 | 1.085 | 1.076 |
| 1:6 | 0.097 | 0.086 | 0.057 | 0.021 | 1.02 | 1.023 | 1.072 | 1.06 |
| 1:18 | 0.084 | 0.065 | 0.052 | 0.018 | 1.043 | 1.045 | 1.028 | 1.056 |
| 1:54 | 0.058 | 0.05 | 0.035 | 0.018 | 0.992 | 0.997 | 0.988 | 1.016 |
| Dilution | C terminal antibody | | | | | | | |
|  | ΔC3 peptide | | | | DR10624 | | | |
|  | 10E10 | 10F6 | **11B1** | 11C1 | 10E10 | 10F6 | **11B1** | 11C1 |
| 1:2 | 0.084 | 0.028 | 0.005 | 0.134 | 1.057 | 0.749 | 1.062 | 1.058 |
| 1:6 | 0.096 | 0.025 | 0.018 | 0.135 | 1.041 | 0.427 | 1.066 | 1.084 |
| 1:18 | 0.068 | 0.015 | 0.015 | 0.111 | 1.067 | 0.148 | 0.972 | 1.026 |
| 1:54 | 0.07 | 0.021 | 0.002 | 0.078 | 0.989 | 0.057 | 0.99 | 0.966 |
| Dilution | C terminal antibody | | | | | | | |
|  | ΔC3 peptide | | | | DR10624 | | | |
|  | 11D12 | 11E9 | 12G11 |  | 11D12 | 11E9 | 12G11 |  |
| 1:2 | 0.024 | 0.069 | 0.179 |  | 1.049 | 1.069 | 1.073 |  |
| 1:6 | 0.037 | 0.053 | 0.2 |  | 1.045 | 1.061 | 1.062 |  |
| 1:18 | 0.04 | 0.055 | 0.242 |  | 1.007 | 1.04 | 1.06 |  |
| 1:54 | 0.031 | 0.052 | 0.192 |  | 1.019 | 0.985 | 1.027 |  |

**Table S6** List of possible truncation fragments of DR10624 from PK study in monkeys

| Species | Theoretical Mass (Da) | Measured Mass (Da) | Difference (ppm) |
| --- | --- | --- | --- |
| Target(S32-G448) | 44521.0 | 44522.0 | 21.8 |
| Target(S32-S478) | 47519.3 | 47520.1 | 16.0 |
| Target(H1-G448) | 47921.7 | 47922.5 | 16.9 |
| Target(H1-S475) | 50540.6 | 50541.0 | 7.9 |
| Unknown peak | / | 50679.5 | / |
| Target (Full-length, H1-S478) | 50919.9 | 50920.5 | 11.8 |

**Table S7** The selectivity result for the ECL method validation (ng/mL)

| Matrix Number | Blank matrix | QC level | | | | | |
| --- | --- | --- | --- | --- | --- | --- | --- |
|  |  | LLOQ (5.00 ng/mL) | | | ULOQ (1250 ng/mL) | | |
|  |  | Calculated  concentration | %CV | Bias% | Calculated  concentration | %CV | Bias% |
| LM-XH23BS0080-03 | BLQ | 5.63 | 0.9 | 12.6 | 1050 | 3.8 | -16 |
| LM-XH23BS0089-06 | BLQ | 5.99 | 0.4 | 19.8 | 1060 | 5.3 | -15.2 |
| LM-XH23BS0110-04 | BLQ | 5.53 | 13.3 | 10.6 | 931 | 9.2 | **-25.5** |
| LM-XH23BS0111-09 | BLQ | 6.22 | 8.6 | 24.4 | 992 | 9.7 | -20.6 |
| LM-XH23BS0123-09 | BLQ | 5.51 | 2.2 | 10.2 | 1120 | 5.5 | -10.4 |
| LM-XH23BS0126-09 | BLQ | 6.56 | 8.1 | **31.2** | 964 | 7.7 | -22.9 |
| LM-XH23BS0127-09 | BLQ | 4.96 | 8.4 | -0.8 | 1120 | 22.5 | -10.4 |
| LM-XH23BS0130-03 | BLQ | 5.49 | 3.6 | 9.8 | 1160 | 7.8 | -7.2 |
| LM-XH23BS0131-02 | BLQ | 5.56 | 6.2 | 11.2 | 982 | 2.5 | -21.4 |
| LM-XH23BS0132-08 | BLQ | 6.11 | 1.6 | 22.2 | 1060 | 2.9 | -15.2 |
|  |  |  |  |  |  |  |  |
| Haemolysed**^1^** matrix 1 | BLQ | 4.57 | 18.4 | -8.6 | 1280 | 3.9 | 2.4 |
| Haemolysed matrix 2 | BLQ | 4.21 | 2 | -15.8 | 1310 | 4.9 | 4.8 |
| Haemolysed matrix 3 | BLQ | 4.3 | 4.9 | -14 | 1350 | 4.2 | 8 |
| Haemolysed matrix 4 | BLQ | 4.03 | 0 | -19.4 | 1310 | 1.6 | 4.8 |
| Haemolysed matrix 5 | BLQ | 4.63 | 1.8 | -7.4 | 1420 | 5.5 | 13.6 |
|  |  |  |  |  |  |  |  |
| Lipaemic**^2^** matrix 1 | BLQ | 5.32 | 24.2 | 6.4 | 1410 | 4.0 | 12.8 |
| Lipaemic matrix 2 | BLQ | 4.73 | 8.5 | -5.4 | 1430 | 4.0 | 14.4 |
| Lipaemic matrix 3 | BLQ | 5.38 | 4.9 | 7.6 | 2020 | **56.0** | **61.6** |
| Lipaemic matrix 4 | BLQ | 4.88 | 4.9 | -2.4 | 1420 | 2.0 | 13.6 |
| Lipaemic matrix 5 | BLQ | 5.46 | 3.9 | 9.2 | 1460 | 7.7 | 16.8 |

**^1^** Haemolysed matrices were obtained by spiking matrix with 2% V/V haemolysed whole blood.

**^2^** Lipaemic matrices were obtained by spiking matrix with to 300 mg/dL.

**Table S8** Dilutional Linearity validation results-from 200,000 ng/mL hook sample

| Sample ID | Dilution factor | Nominal Concentration  (ng/mL) | Mean Result  (ng/mL) | % CV | % Bias | Final Concentration  (ng/mL) |
| --- | --- | --- | --- | --- | --- | --- |
| Hook1-1 | 1 | 200000 | ALQ |  |  | ALQ |
| Hook2-1 | 1.6 | 125000 | ALQ |  |  | ALQ |
| Hook3-1 | 25 | 8000 | ALQ |  |  | ALQ |
| DL-1 | 250 | 800 | 786 | 12.9 | -1.5 | 197000 |
|  |  |  | 819 | 13.0 | 2.5 | 205000 |
|  |  |  | 839 | 5.5 | 5.0 | 210000 |
|  |  |  | 730 | 0.7 | -8.5 | 183000 |
|  |  |  | 768 | 3.8 | -4.0 | 192000 |
| DL-2 | 500 | 400 | 386 | 7.6 | -3.5 | 193000 |
|  |  |  | 388 | 0.9 | -3.0 | 194000 |
|  |  |  | 444 | 6.0 | 11.0 | 222000 |
|  |  |  | 363 | 5.9 | -9.0 | 182000 |
|  |  |  | 391 | 4.8 | -2.0 | 196000 |
| DL-3 | 1000 | 200 | 176 | 2.9 | -12.0 | 176000 |
|  |  |  | 182 | 0.7 | -9.0 | 182000 |
|  |  |  | 203 | 11.4 | 1.5 | 203000 |
|  |  |  | 179 | 1.6 | -10.5 | 179000 |
|  |  |  | 194 | 6.3 | -3.0 | 194000 |
| DL-4 | 2000 | 100 | 90.9 | 1.5 | -9.0 | 182000 |
|  |  |  | 98.6 | 4.7 | -1.5 | 197000 |
|  |  |  | 99 | 7.0 | -1.0 | 198000 |
|  |  |  | 92.4 | 6.0 | -7.5 | 185000 |
|  |  |  | 101 | 0.4 | 1.0 | 202000 |
| DL-5 | 4000 | 50 | 46.9 | 3.6 | -6.0 | 188000 |
|  |  |  | 50.7 | 4.9 | 1.5 | 203000 |
|  |  |  | 48.9 | 0.5 | -2.0 | 196000 |
|  |  |  | 47.5 | 5.7 | -5.0 | 190000 |
|  |  |  | 49.9 | 6.1 | 0 | 200000 |
| DL-6 | 80000 | 2.5 | BLQ | - | - | BLQ |
|  |  |  | BLQ | - | - | BLQ |
|  |  |  | BLQ | - | - | BLQ |
|  |  |  | BLQ | - | - | BLQ |
|  |  |  | BLQ | - | - | BLQ |

**Table S9** Comparison between the requirement of ICH 10 and the validation results in our study.

| Parameters | ICH M10 | Results in our study |
| --- | --- | --- |
| Selectivity | at least 10 normal individual matrices. | 9 in 10 individuals met the criteria |
|  | at least 5 haemolysed samples. | 5 in 5 individuals met the criteria |
|  | at least 5 lipaemic samples. | 4 in 5 individuals met the criteria |
|  | The accuracy of 80% test individuals within ±25% at the LLOQ and within ±20% at the high QC. | All met the criteria |
| Accuracy and Precision | The QCs should be prepared at five concentration levels: LLOQ, low QC (LQC), medium QC (MQC), high QC (HQC) and ULOQ. | Five QCs were prepared including LLOQ, LQC, MQC, HQC and ULOQ |
|  | Within-run and between-run precision of the QC concentrations determined at each level should not exceed 20%, except at the LLOQ and ULOQ, where it should not exceed 25%. | Precision of QCs were within 9.4%~13.9% for intra and inter assays |
|  | The overall within-run and between-run accuracy at each concentration level should be within ±20% of the nominal values, except for the LLOQ and ULOQ, which should be within ±25% of the nominal. | Accuracy of QCs were within -4.8%~14.7% for intra and inter assays |
|  | The total error should not exceed 30% (40% at LLOQ and ULOQ). | Total errors of QCs were within 14.4%~25.2% |
| Dilutional linearity | The calculated mean concentration for each dilution should be within ±20% of the nominal concentration after correction for dilution and the precision should not exceed 20%. | Up to 1:4000 diluted in serum matrix met the requirement |
| Hook effect | No hook effect observed above ULOQ. | No hook effect observed up to 200,000 ng/mL far above ULOQ (1250 ng/mL) |
| Stability | The investigation of stability should cover bench top (short-term) stability at room temperature or sample preparation temperature and freeze-thaw stability. In addition, long-term stability should be studied. | Stable if freezed at nominal -80℃ and thawed at RT: 6 cycles |
|  |  | Stable if stored at 2-8℃ and RT: up to 24 h 17 mins |
|  |  | Stable if stored at nominal -80℃: 195 days |

**Figure S1.** Characterization of DR10624 and variants by SDS-PAGE and HPLC-SEC.

**A.** SDS-PAGE of DR10624, DR10624 ΔN2 and DR10624 ΔC4. The molecular weight (MW) of the intact DR10624 is approximately 101.8 kDa.

**B**. Purity analysis of DR10624 and its variants by HPLC-SEC (S200 column, Agilent). DR10624, DR10624 ΔN2, and DR10624 ΔC4 each showed >95% purity, with retention times of 8.529, 8.632, and 8.669 min, respectively.

**Figure S2** Flowchart for generation of DR10624 terminus specific antibodies

**Figure S3** Characterization of DR10624 terminus antibodies by SDS-PAGE and HPLC-SEC.

**A.** Reduced SDS-PAGE of DR10624 terminus antibodies. MW, molecular weight.

**B**. Purity analysis of DR10624 terminus antibodies by HPLC-SEC (S200 column, Agilent). All the antibodies showed >95% purity, except 14B3 (94.31%).

**Figure S4** Binding specificities of N terminal and C terminal antibodies.

**A and B.** The binding activities of N terminal antibodies 4G7 and 14B3 to BSA conjugated ΔN2 peptide and human IgG mixture, and an isotype antibody was used as negative control (NC).

**C and D.** The binding activities of C terminal antibodies 1A12, 7A8 and 11B1 to BSA conjugated ΔC3 peptide and human IgG mixture, and an isotype antibody was used as negative control (NC).

**Figure S5.** Antibody pairs for the quantification of DR10624.

**A.** Antibody pairs with 14B3 coated as capture antibody and biotinylated 1A12, 7A8 and 11B1 as detection antibody, respectively.

**B.** Antibody pairs with 7A8 coated as capture antibody and biotinylated 4G7, 14B3 as detection antibody, respectively.

**C.** Antibody pairs with 11B1 coated as capture antibody and biotinylated 4G7, 14B3 as detection antibody, respectively.

**Figure S6.** Identification of truncation fragments by LC-MS under reduced condition.

**Figure S7.** Relative potency of serum samples collected at different time points.

PK samples collected at 24 h, 96 h, and 168 h post-administration were evaluated for agonistic activities on GLP-1R, GCGR, and FGF21R. The RLU readouts at 24 h for each corresponding assay were normalized to 100%.

**Figure S8.** Typical standard curve of DR10624 standards (n=2).

A typical standard curve of DR10624 included concentrations in human serum of 2.50 (anchor point), 5.00, 10.0, 25.0, 80.0, 200, 500, 750, 1000, 1250 ng/mL.

**Figure S9.** The binding specificities of 4G7 and 1A12

A. The binding specificity of N terminal antibody 4G7 to native GLP-1, native GCG, native FGF21, DR10624 and N terminal peptide of DR10624.
B. The binding specificity of C terminal antibody 1A12 to native GLP-1, native GCG, native FGF21, DR10624 and N terminal peptide of DR10624.
